# Effects of infection by *Turnip mosaic virus* on the population growth of generalist and specialist aphid vectors on turnip plants

**DOI:** 10.1371/journal.pone.0200784

**Published:** 2018-07-17

**Authors:** Shuhei Adachi, Tomoki Honma, Ryosuke Yasaka, Kazusato Ohshima, Makoto Tokuda

**Affiliations:** 1 The United Graduate School of Agricultural Sciences, Kagoshima University, Kagoshima, Japan; 2 Laboratory of Systems Ecology, Faculty of Agriculture, Saga University, Saga, Japan; 3 Laboratory of Plant Virology, Faculty of Agriculture, Saga University, Saga, Japan; Oklahoma State University, UNITED STATES

## Abstract

Recent studies have revealed that relationships between plant pathogens and their vectors differ depending on species, strains and associated host plants. Turnip mosaic virus (TuMV) is one of the most important plant viruses worldwide and is transmitted by at least 89 aphid species in a non-persistent manner. TuMV is fundamentally divided into six phylogenetic groups; among which Asian-BR, basal-BR and world-B groups are known to occur in Japan. In Kyushu Japan, basal-BR has invaded approximately 2000 and immediately replaced the predominant world-B virus group. To clarify the relationships between TuMV and vector aphids, we examined the effects of the TuMV phylogenetic group on the population growth of aphid vectors in turnip plants. The population growth of a generalist aphid, *Myzus persicae*, was not significantly different between non-infected and TuMV-infected treatments. The population growth of a specialist aphid, *Lipaphis erysimi*, was higher in TuMV-infected plants than non-infected ones. Similar results were obtained in experiments using world-B and basal-BR groups of TuMV. Therefore, we conclude that *L*. *erysimi* is more mutualistic with TuMV than *M*. *persicae*, and differences in TuMV phylogenetic groups do not affect the growth of aphid vectors on turnip plants.

## Introduction

Many plant pathogenic viruses expand their infection range with the aid of sucking insect vectors such as aphids, whiteflies and leafhoppers [1, 2]. Transmission by these insects is divided into persistent and non-persistent manners. In persistent viruses, vectors retain viruses inside their bodies for a relatively long time and continue the infective state through molting. Therefore, mutualistic relationships evolve easily between the virus and the vector [3]. In non-persistent viruses, vectors acquire and retain viruses only in their stylets for a relatively short period of time, and easily lose viruses after molting or feeding on an uninfected plant [4].

Recent studies have revealed that relationships between plant pathogens and their vectors differ depending on species, strains and associated host plants [5–10]. For example, a study [9] showed that tomato yellow leaf curl virus infecting tomato plants positively affected the growth performance of the vector, biotype Q of *Bemisia tabaci*, but negatively affected biotype B. This difference may be the cause for changes in dominant whitefly biotypes by Q in various localities in China. Although the majority of agricultural crop viruses causing severe economic damage are transmitted in a non-persistent manner especially by aphids [11], relationships between vectors and non-persistent viruses have seldom been investigated because of the extremely short contact time between the virus and the vector [3].

Turnip mosaic virus (TuMV) is one of the most important plant viruses worldwide and is transmitted by at least 89 aphid species in a non-persistent manner [12]. TuMV is fundamentally divided into six phylogenetic groups [13–14]. Of these groups, the Asian-BR, basal-BR and world-B groups are known to occur in Japan. In Japan, basal-BR invaded the Kyushu Region in approximately the year 2000 and immediately overcame the predominant world-B group, but the cause of this replacement remains unknown [15]. A previous study [16] reported that the reproductive performance of the generalist aphid vector *Myzus persicae* (Sulzer) increased in *Nicotiana benthamiana* Domin (Solanaceae) and *Arabidopsis thaliana* L. (Heyn.) (Brassicaceae) infected with TuMV. However, the effects of TuMV infection on the reproductive performance of vector aphids has not been investigated in brassicaceous crops, which suffer severe economic damages from TuMV in Japan [17–19]. In addition, these vegetables are infested by both generalist aphid vectors including *M*. *persicae* and specialist aphid vectors [20–21]. Therefore, clarification of interactions among brassicaceous crops, phylogenetic groups of TuMV and vector aphids is important for establishing management measures for these agricultural pests and pathogens.

The purpose of this study was to clarify whether the relationships between TuMV and vector aphids are different among phylogenetic groups of TuMV and vector aphid species. To assess these relationships, we surveyed the population growth of two aphid vectors, generalist aphid *M*. *persicae* and specialist aphid *Lipaphis erysimi* (Kaltenbach), on TuMV-infected and non-infected Japanese turnip plants using isolates of world-B and basal-BR phylogenetic groups of TuMV.

## Materials and methods

### Aphid populations

Colonies of *M*. *persicae* and *L*. *erysimi* were collected from *Brassica napus* L. in Saga City, Saga, Japan in 2014 (Saga strains: 33°14’12.2”N, 130°17’47.2”E). Because the Saga strain of *L*. *erysimi* expired during the course of this study, another colony of *L*. *erysimi* was collected from Japanese radish in Ogi City, Saga, Japan in 2014 (Ogi strain: 33°16’40.6”N, 130°13’06.6”E). Saga strains were acquired from Honjo Park and did not require specific permission to collect. Ogi strain was acquired from a private land and we obtained the owner’s permission to conduct the study on the site. Aphids were any protected or endangered species present in these fields. These colonies were continuously reared on turnip plants (*Brassica rapa* cv. “Hakatasuwari”) in an insect chamber at 25°C and 16L:8D photoperiodic conditions.

### Virus isolates and inoculation of source plants

The TuMV isolates of C42J [22] from *B*. *rapa* and OGI11X2 from *Raphanus sativus* L. were used as representatives of world-B and basal-BR phylogenetic groups, respectively [23]. Both isolates were inoculated onto *Chenopodium quinoa* Wild. and serially cloned one time through single lesions. Each virus was propagated in *B*. *rapa* cv. Hakatasuwari or *N*. *benthamiana* plants. To prepare the virus source plants, each TuMV isolate was separately inoculated onto young seedlings of *N*. *benthamiana* at the 1-true leaf developmental stage by mechanical sap inoculation [22]. Then, seedlings were kept for at least ten days in a glasshouse at 25°C until use as inoculation source plants.

### Reproductive performance experiments on TuMV-infected turnips

Virus source plants infected with world-B and basal-BR isolates were homogenized in 0.01 M potassium phosphate buffer (pH 7.0) and mechanically inoculated onto *B*. *rapa* cv. Hakatasuwari plants at the 1-true leaf stage. Healthy plants were treated with the same buffer solution at the same growth stage and used as non-infected controls. Inoculated plants were kept for ten days in a glasshouse at 25°C, and then used for experiments.

To assess the effects of TuMV infection on aphid reproduction, five first instar nymphs of *M*. *persicae* or *L*. *erysimi* were placed on world B-infected and non-infected turnip plants. Similarly, one first instar nymph (due to low numbers of first instars) of *M*. *persicae* or *L*. *erysimi* was placed on basal-BR-infected and non-infected turnip plants. All aphids on each plant were counted after six days. Nine or ten replications were performed for each treatment and control. The Saga strain of *L*. *erysimi* was used in the experiments with the world-B group and the Ogi strain in the experiments with the basal-BR group. The numbers of aphids between in infected and non-infected turnip plants were analyzed using a Student’s t-test.

## Results

### Reproductive performance experiments on TuMV-infected turnips

**Fig 1:**
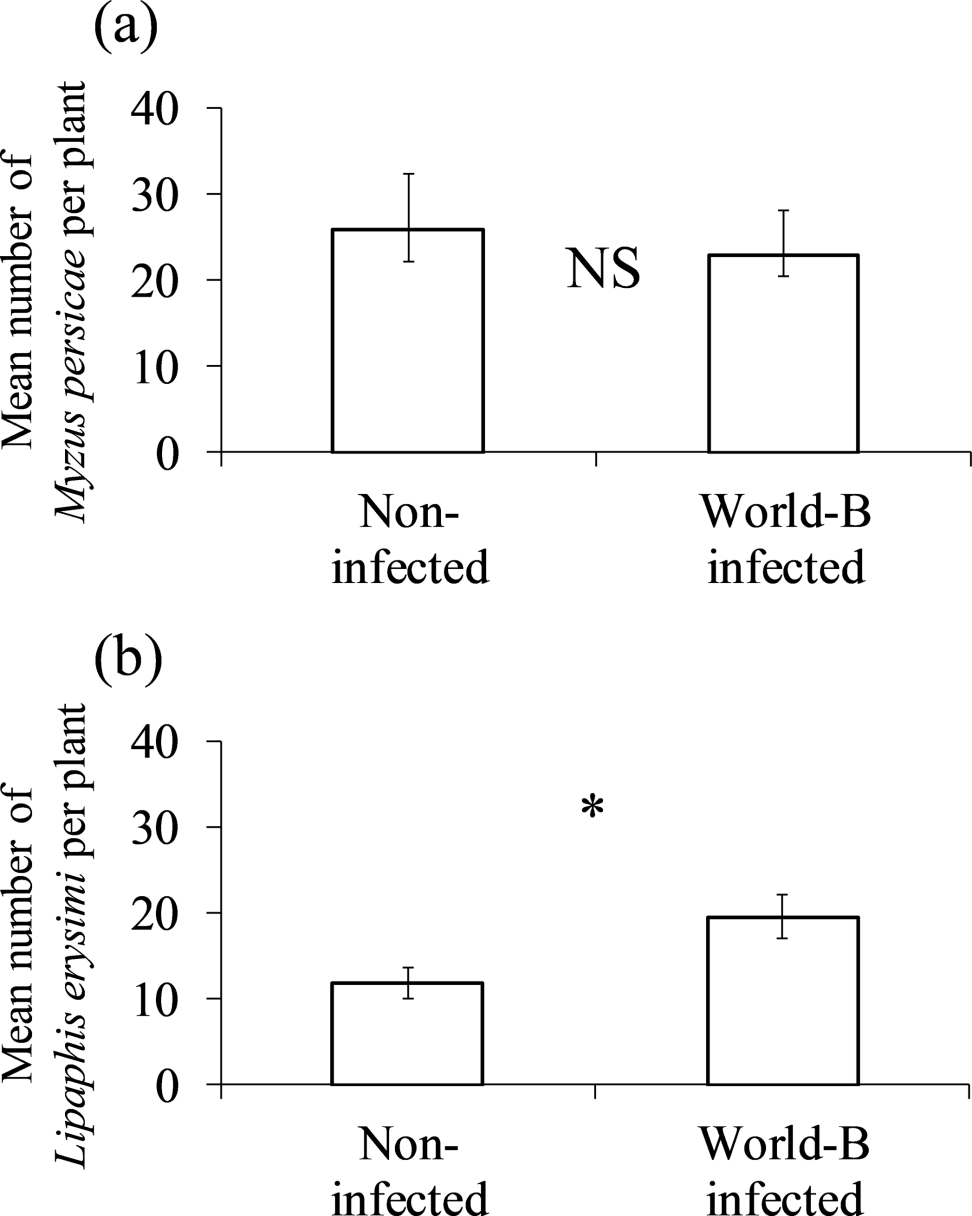
**Mean number of (a) *M*. *persicae* and (b) *L*. *erysimi* on non-infected and world-B infected turnip plants.** Vertical bars represent ±1 standard error of the mean. Asterisk and “NS” indicate significant and non-significant differences, respectively, between non-infected and world-B infected turnip plants (p < 0.05).

The number of *M*. *persicae* on turnip plants was not significantly different between non-infected and world-B infected treatments (t = 0.71, p = 0.49, Student’s t-test; Fig 1A). Meanwhile, the number of *L*. *erysimi* was significantly higher on world-B infected turnip plants than non-infected ones (t = -2.45, p < 0.05, Student’s t-test; Fig 1B).

**Fig 2:**
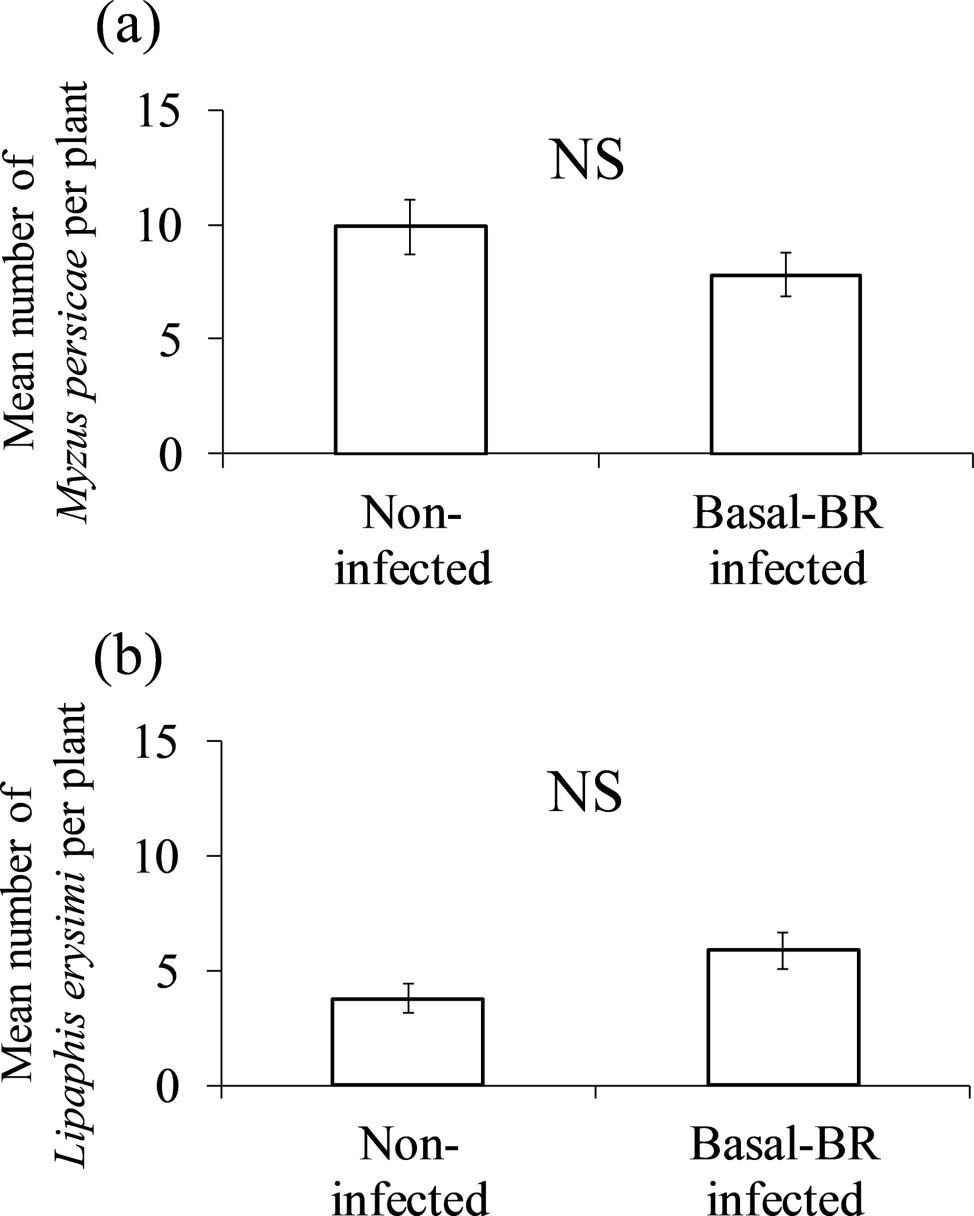
**Mean number of (a) *M*. *persicae* and (b) *L*. *erysimi* on non-infected and basal-BR infected turnip plants.** Vertical bars represent ±1 standard error of the mean. “NS” indicates no significant difference between non-infected and basal-BR infected turnip plants (p < 0.05).

Similarly, no significant differences were detected in the number of *M*. *persicae* on turnip plants between non-infected and basal-BR infected treatments (t = 1.41, p = 0.18, Student’s t-test; Fig 2A), while the number of *L*. *erysimi* tended to be higher on basal-BR infected turnip plants than on non-infected ones (t = -2.10, p = 0.052, Student’s t-test; Fig 2B).

## Discussion

In this study, we examined the population growth of two aphid vectors of TuMV on turnip plants infected by world-B and basal-BR phylogenetic groups of TuMV. The population growth of *M*. *persicae* was not promoted by TuMV infection, regardless of phylogenetic group. This is consistent with results shown by a previous study [24], in which the reproductive performance of *M*. *persicae* did not change on TuMV-infected *N*. *tabacum*. However, another previous study reported that the performance of *M*. *persicae* on *N*. *benthamiana* and *A*. *thaliana* increased with TuMV infection [16]. The previous report [24] demonstrated that, under the presence of *M*. *persicae*, a viral protein, Nla-Pro, localized to a vacuole in *N*. *benthamiana* but not in *N*. *tabacum*, and concluded that this phenomenon critically affects the increase of *M*. *persicae* reproductive performance in *N*. *benthamiana*. Further studies are needed to clarify whether the localization of Nla-Pro is observed or not in our system.

In contrast to *M*. *persicae*, the population growth of *L*. *erysimi* significantly increased on world-B infected plants and tended to increase on basal-BR infected plants, when compared to non-infected treatments. As mentioned earlier, *L*. *erysimi* is a specialist aphid associated only with Brassicaceae, while *M*. *persicae* is a generalist with a broad host range of over 40 plant families [25]. Therefore, the former species may be better adapted to Brassicaceae plants with TuMV. In addition, our results suggest that not only differences in plant species but also combinations of vectors and host plants are important factors affecting the performance of vector aphids.

In the present study, effects of TuMV infection on aphid reproductive performance were similar between world-B and basal-BR groups, suggesting that other factors are related to the replacement of the TuMV phylogenetic group world-B with the basal-BR in Kyushu. A previous study [26] showed that host range and infection efficiency are different among TuMV phylogenetic groups. Therefore, infection capacities related to Japanese crops and wild host plants may have caused the replacement in Kyushu. Another possibility is co-infection with other plant viruses may have affected the dominance of TuMV phylogenetic groups because mixed infections of TuMV and other viruses are often confirmed in field situations [12, 17–18]. Further studies are needed to clarify the factors causing the replacement of TuMV phylogenetic groups in Kyushu.

In summary, this study shows the relationships between TuMV and its aphid vectors differ between *L*. *erysimi* and *M*. *persicae*, but do not appear to differ between the two phylogenetic groups of TuMV, world-B and basal-BR.
